# Systems Biology Approaches and Precision Oral Health: A Circadian Clock Perspective

**DOI:** 10.3389/fphys.2019.00399

**Published:** 2019-04-16

**Authors:** Henry A. Adeola, Silvana Papagerakis, Petros Papagerakis

**Affiliations:** ^1^Hair and Skin Research Laboratory, Division of Dermatology, Department of Medicine, Faculty of Health Sciences and Groote Schuur Hospital, University of Cape Town, Cape Town, South Africa; ^2^Department of Oral and Maxillofacial Pathology, Faculty of Dentistry, University of the Western Cape and Tygerberg Hospital, Cape Town, South Africa; ^3^Laboratory of Oral, Head & Neck Cancer–Personalized Diagnostics and Therapeutics, Division of Head and Neck Surgery, Department of Surgery, University of Saskatchewan, Saskatoon, SK, Canada; ^4^College of Dentistry, University of Saskatchewan, Saskatoon, SK, Canada

**Keywords:** clock genes, circadian disruption, systems biology, pathology, head and neck cancer, Sjögren's syndrome, precision medicine and dentistry, patient oriented research

## Abstract

A vast majority of the pathophysiological and metabolic processes in humans are temporally controlled by a master circadian clock located centrally in the hypothalamic suprachiasmatic nucleus of the brain, as well as by specialized peripheral oscillators located in other body tissues. This circadian clock system generates a rhythmical diurnal transcriptional-translational cycle in clock genes and protein expression and activities regulating numerous downstream target genes. Clock genes as key regulators of physiological function and dysfunction of the circadian clock have been linked to various diseases and multiple morbidities. Emerging omics technologies permits largescale multi-dimensional investigations of the molecular landscape of a given disease and the comprehensive characterization of its underlying cellular components (e.g., proteins, genes, lipids, metabolites), their mechanism of actions, functional networks and regulatory systems. Ultimately, they can be used to better understand disease and interpatient heterogeneity, individual profile, identify personalized targetable key molecules and pathways, discover novel biomarkers and genetic alterations, which collectively can allow for a better patient stratification into clinically relevant subgroups to improve disease prediction and prevention, early diagnostic, clinical outcomes, therapeutic benefits, patient's quality of life and survival. The use of “omics” technologies has allowed for recent breakthroughs in several scientific domains, including in the field of circadian clock biology. Although studies have explored the role of clock genes using circadiOmics (which integrates circadian omics, such as genomics, transcriptomics, proteomics and metabolomics) in human disease, no such studies have investigated the implications of circadian disruption in oral, head and neck pathologies using multi-omics approaches and linking the omics data to patient-specific circadian profiles. There is a burgeoning body of evidence that circadian clock controls the development and homeostasis of oral and maxillofacial structures, such as salivary glands, teeth and oral epithelium. Hence, in the current era of precision medicine and dentistry and patient-centered health care, it is becoming evident that a multi-omics approach is needed to improve our understanding of the role of circadian clock-controlled key players in the regulation of head and neck pathologies. This review discusses current knowledge on the role of the circadian clock and the contribution of omics-based approaches toward a novel precision health era for diagnosing and treating head and neck pathologies, with an emphasis on oral, head and neck cancer and Sjögren's syndrome.

## Introduction

Precision health has emerged as a novel approach for disease prevention, diagnosis, and treatment that accounts for individual variability in genes, environment, and lifestyle; aiming to tackle interpatient and disease heterogeneity to ensure accurate diagnostic and tailored therapeutic approaches which take into account the individual as a whole. Precision health approaches require integrating comprehensive molecular, genetic, burden of disease(s) aspects and exposure to risks into a holistic profile of a given individual that will allow for a customized and comprehensive care plan to improve individual health outcomes. Multi-omics and system biology approaches are becoming the golden standards to pave the road for precision health. However, correlation-focused big data analytics are not yet standardized for data collection, interpretation and objectivity, thus efforts are hampered by biases and contradictory results, which often ignore causality and individual complexity (Bottles and Begoli, 2014).

As an example for head and neck, Rai et al. reported a review analysis of to-date studies based on omics-approaches (total of 81, including genomics, proteomics, transcriptomics and metabolomics) applied to differentiate among oral squamous cell carcinomas, oral premalignant lesions (such as oral leucoplakia, oral lichen planus, oral erythroplakia, oral submucous fibrosis) and normal cases. This study highlighted the advanced ability of omics technologies to screen for early changes in DNA, RNA, protein, and metabolite expression, in support of a much needed early detection of oral cancer through comprehensive molecular profiling, to overcome the current diagnostic delays. Worldwide, the vast majority of oral cancer cases are diagnosed at late/advanced stages and this is responsible for the poor oral cancer survival rate, one of the lowest among other most common cancers types (Rai et al., 2018). Additional studies reviewed other available databases (e.g., COSMIC, database for somatic mutations in cancer) to compile a list of the most frequently mutated genes in head and neck cancer (Agarwal, 2016), highlighting the need for complementary multi-omics approaches.

Multilayered omics-based analyses have emerged only recently, along with open source big data analytic platforms, databases and repository of health information, along with data mining algorithms, but more often the effects of the circadian clock regulatory events (as can be found in circadiOmics) are ignored, and individual oral health morbidities even more so. In flagrant contrast, the burden of oral health remains extremely high worldwide. Poor oral and dental health is universally recognized as having a profound effect on general health, well-being and quality of life (Petersen et al., 2005), while the oral conditions are the most common conditions of humankind (FDI, 2015).

Taking into account that 43% of all protein coding genes shown circadian rhythms in transcription (Zhang et al., 2014), it is becoming evident that circadian clock regulatory effects on gene and protein expression need to be taken in consideration in parallel with the multi-omics analyses to achieve an authentic level of precision health. Indeed, disruptions of the circadian clock have been associated with nearly every major human disease, and many of the most commonly-used medications target circadian genes (Takahashi et al., 2008; Zhang et al., 2014). Although the clinical relevance of the linkage of clock genes and circadian disruption to disease pathogenesis is yet to be fully elucidated, taking in consideration circadian variations in multi-omics analyses, recently called circadiOmics (Ceglia et al., 2018) is a critical step toward unbiased precision health. Understanding the gaps in the role of the circadian rhythm/clock gene in disease pathogenesis has the potential to open new avenues for targeted therapies of clock gene-controlled diseases. Recognizing the critical importance of circadian clock in precision health is in alignment with the recent acknowledgment of the clock genes' major biological significance, the 2017 Nobel Prize in Physiology or Medicine was jointly awarded to Jeffrey C. Hall, Michael Rosbash and Michael W. Young for their discoveries of the molecular mechanisms controlling the circadian rhythms (https://www.nobelprize.org/nobel_prizes/medicine/laureates/2017/press.html). This further acknowledges the profound clinical relevance of the circadian clock mechanism and highlights the need for a better understanding of the implications of clock genes and circadian disruption to disease pathogenesis and related therapeutics (Bass, 2017).

We have recently shown that circadian clock is a major determinant of craniofacial tissues cell homeostasis, regulating proliferation and differentiation of salivary glands (Zheng et al., 2012), dental cells (Athanassiou-Papaefthymiou et al., 2011; Zheng et al., 2011, 2013) and oral epithelium (Zheng et al., 2011; Papagerakis et al., 2014); linking circadian clock disruption with major oral, head and neck pathologies, such as oral cancer and Sjögren syndrome (Mitsiadis et al., 2014; Papagerakis et al., 2014; Matsumoto et al., 2016; Zagni et al., 2017). In parallel, there is an increasing interest in applying multi-omics and system biology approaches to studying head and neck pathologies, thus considering oral health as an integral part of precision health initiatives (Niehr et al., 2018; Rai et al., 2018). Hence, this review focuses on the importance of circadian clock in the system biology approaches applied to oral precision health as well as on the high throughput omics-based techniques applications toward identifying novel targetable molecular circadian alterations in head and neck pathologies, with an emphasis on oral, head and neck carcinoma and Sjögren Syndrome.

## Circadian Clock, Oral Tissues Homeostasis, and Head and Neck Pathologies

### The Circadian Clock Mechanism: Brief Introduction to Circadian Biology

#### Clock Genes, Circadian Rhythms, and Circadian Synchrony

Across all phyla, daily physiological and behavioral rhythms are controlled by endogenous circadian clocks (Merrow et al., 2005). The circadian system is a vital metabolic and behavioral integrator that synchronizes rotation of the Earth on its axis with physiological processes (Marcheva et al., 2009). In anticipation of daily events, circadian cells that contain intrinsic rhythms are synchronized to each other and to environmental cues, such as the light/dark cycle (Xu et al., 2011). Circadian synchrony has been described as a situation where cells or organisms exhibit the same daily (near 24-h period) cycle (Herzog et al., 2015; Husse et al., 2015). This has been described in unicellular organisms, such as dinoflagellates, cyanobacteria; as well as in metazoans including fungi, algae, plants, flies, birds, rodents and humans (Bell-Pedersen et al., 2005; Herzog et al., 2015). In a marching band, instrumentalists, synchronize their pace to their neighbors and perform with the same period, albeit they may be phase delayed in their sequence of arrival (Herzog et al., 2015). Similarly, circadian synchrony may involve oscillators that undergo frequency entrainment without the requirement of peaking together (period synchrony), or they may involve oscillators that establish a locked, stable, unique phase relationship (phase synchrony) (Herzog et al., 2015). Unicellular organisms utilize stand-alone clocks that are capable of generating 24-h rhythms for various processes, but higher organisms can establish and coordinate multiple biological rhythms by partitioning clock gene functions; in both scenarios, robust circadian rhythms of biological activity and gene expression are generated under the temporal synchronization of a multi-oscillator system (Bell-Pedersen et al., 2005).

#### Peripheral and Central Clocks

There are different models that describe the relationship between the central and peripheral clocks, such as the “master-slave,” “orchestra,” and “federate” models (Dibner et al., 2010; Buhr and Takahashi, 2013; Pilorz et al., 2018). The canonical “master-slave” model hypothesized that in humans (as in many other metazoans), the master/central clock situated in the suprachiasmatic nuclei (SCN) of the hypothalamus (Gillette and Tischkau, 1999; Reghunandanan and Reghunandanan, 2006; Rosenwasser and Turek, 2015) controls peripheral clocks that are present in other regions of the body (Papagerakis et al., 2014). SCN clock-gene rhythms are more rapidly entrained in response to light than they are in peripheral oscillators (Yamazaki et al., 2000; Bell-Pedersen et al., 2005; Duffy and Czeisler, 2009; Papagerakis et al., 2014). In this model, the peripheral clocks can be entrained by the central clock, or they can synchronize to the environment, or to one another, to harmonize daily physiological rhythms, such as sleeping/waking, feeding/fasting, metabolism, hormonal levels, temperature, and gene expression (Baggs and Hogenesch, 2010; Froy and Miskin, 2010; Feng and Lazar, 2012; Papagerakis et al., 2014). The (orchestra) model alludes to the independence and the potential for direct external influence of the peripheral clocks, based on *in vitro* and explant rhythm study evidences (Balsalobre et al., 2000; Yoo et al., 2005; Dibner et al., 2010). These studies demonstrated that the central clock may be acting as an orchestra conductor; while each peripheral clock acts as a musician (Pilorz et al., 2018). Although, direct external stimuli might fine tune peripheral rhythms, the peripheral clocks still rely on entrainment from the SCN pacemaker cues. The observation that genetic ablation of SCN pacemaker function did not result in loss of light/dark entrainment in mice, led to the development of the “federate model,” which hypothesizes that SCN function on peripheral clocks is mostly important under non-rhythmic environmental conditions (Husse et al., 2014; Izumo et al., 2014). Our group has provided evidence of the peripheral clock in various organs and tissues in the oral cavity, such as salivary glands and teeth (Papagerakis et al., 2014).

#### Transcription-Translation Feedback Loops (TTFL) of the Clock Genes

Because it is well-established that most organisms utilize interlocked transcription-translation feedback loops (TTFLs), the relevance of the circadian rhythm and its underlying molecular physiology have been broadly analyzed by systems and computational biologists (Novak and Tyson, 2008; Qin et al., 2010; Brown et al., 2012; Rey and Reddy, 2013; McClung, 2014; Papagerakis et al., 2014; Ki et al., 2015; Hurley et al., 2016). The mammalian circadian rhythm is influenced by two important negative feedback loops in the TTFL network (Barinaga, 2000; Papagerakis et al., 2014; Pett et al., 2016). In the first negative feedback loop, Period (PER) and Cryptochrome (CRY) protein form a complex casein kinase I, which then undergoes nuclear translocation and inhibits BMAL1/CLOCK heterodimers, which results in the suppression of Cry, Per and Rev-Erbα transcription. In the second negative feedback loop, CLOCK and BMAL1 activators are expressed, leading to their own transcription inactivation via Rev-Erbα (Papagerakis et al., 2014). A stoichiometric balance between activators, such as PER and CRY and repressors, such as NPAS2 (Neuronal PAS Domain Protein 2, which is paralogous to CLOCK, both key proteins involved in the maintenance of circadian rhythms in mammals) and BMAL1/CLOCK has been identified as pivotal to sustained circadian oscillations (Kim and Forger, 2012). An imbalance in the circadian rhythm can, therefore, lead to various diseases *via* alterations in gene dosage in the TTFL (Lee et al., 2011).

### Circadian Clock and Health

#### Circadian Disruption and Metabolism

Over the past decades, interconnections between metabolic systems and circadian rhythm have received increased attention in clinical studies (Marcheva et al., 2009). There are experimental and genetic evidences for crosstalk between metabolic transcription networks and the circadian system. Circadian clock genes have been found to be linked to metabolic factors and nutrient signaling pathways (Bass and Takahashi, 2010; Schmutz et al., 2010). For instance, Δ19 mutations in the Clock gene has been shown to cause hyperleptinemia, hyperlipidemia, hypoinsulinemia, and hyperglycemia (Turek et al., 2005). Loss of BMAL1 has also been shown to result in impaired adipogenesis, loss of locomotor activity and altered rhythm (Rudic et al., 2004; Shimba et al., 2005; Lamia et al., 2008). Deficiency of CRY (1 and 2) has been shown to be responsible for variations in circulating growth hormone patterns, impairment of growth and alterations in the steroidogenic/lipogenic pathways (Bur et al., 2009). In addition, alterations in bone density and loss of glucocorticoid rhythmicity has been shown to result from deficiency in PER2 (Fu et al., 2005; Yang et al., 2009). Hassan et al. identified perturbation of circadian rhythms in bone marrow stromal cells by titanium biomaterials that are used for dental and orthopedic implants (Hassan et al., 2017). They identified suppression of PER1 expression and an increase in NPAS2 when bone marrow mesenchymal stromal cells were cultured on titanium biomaterials; suggesting that the local aberrant peripheral circadian rhythm may be important for the bio-integration of the titanium into bone (Hassan et al., 2017). Polymorphisms in the mammalian core proteins, such as BMAL1 and CLOCK have been identified to be linked to certain features of the metabolic syndrome, which is associated with the risk of developing cardiovascular disease and type 2 diabetes (Woon et al., 2007; Scott et al., 2008; Sookoian et al., 2008; Maury et al., 2010). It has been hypothesized that circadian mechanisms may influence pro-neurodegenerative activities because aggregation of soluble neuronal proteins due to circadian disruption may lead to proteostasis and may play a vital role in the pathophysiology of neurodegenerative diseases (Hastings and Goedert, 2013).

#### Circadian Disruption and Multiple Morbidities

Perturbation of the internal clock system has been found comorbid with various medical conditions, such as diabetes mellitus, obesity, thrombosis, neurodegenerative diseases (Musiek, 2015), cardiovascular disease (Takeda and Maemura, 2015), cancer (Hu et al., 2014), psychiatric disorders (Lamont et al., 2007), autoimmune and inflammatory diseases (Maury et al., 2010) and sleep disorders (Takahashi et al., 2008; Zhu and Zee, 2012; Jones et al., 2013). Allergic conditions, such as asthma is strongly interlinked with diurnal variations in circadian rhythm (Bass, 2017). A growing number of epidemiological studies stratifying the participants by sex, occupation, geographic location and duration of sleep pattern disruption, have examined associations between night shift work and the risk of various types of cancers (including multiple primary cancers) and various diseases that affect individuals' life span (Bass and Lazar, 2016; Yuan et al., 2018).

### Circadian Clock Disruption and Head and Neck Development and Pathologies

Circadian clock genes and their expression have been reported in head and neck tissues (Simmer et al., 2010; Zheng et al., 2011, 2012, 2013). It has been observed that incremental growth patterns and mineralization of dental tissue is under the influence of various complex biological clock interactions (Simmer et al., 2010). Ameloblasts differentiation regulatory pathways can be characterized by an in-depth understanding of circadian mechanism (Athanassiou-Papaefthymiou et al., 2011; Zheng et al., 2013). Even though the role of circadian clocks in the oral epithelium is poorly understood, circadian rhythms and clock genes expression have been detected in basal cells of oral epithelium, including palatal and junctional epithelia, the epithelial rests of Malassez surrounding dental roots; furthermore, expression of various clock genes were also detected in ameloblast and odontoblast cells, dental pulp cells, periodontal dental ligament cells, osteoblast and osteoclasts in the alveolar bone (Zheng et al., 2011; Papagerakis et al., 2014). Similarly, clock gene expression has been demonstrated both in kidney and salivary gland tissues (Zheng et al., 2012), and their expression may affect function and saliva composition (Dawes, 1972). Furthermore, the kidney which has some similar physiological function to the salivary glands (such as producing ultrafiltrates of blood-urine and saliva, respectively) has been shown to be under the control of circadian clock mechanisms (Stow and Gumz, 2011). Upregulation of aquaporin genes associated with fluid movement have been reported both in the salivary glands, as well as in the kidneys (Ishikawa and Ishida, 2000; Maeda et al., 2008; Delporte et al., 2016). An abnormal expression of clock genes can alter aquaporin expression which may result in a change in salivary flow rate (Papagerakis et al., 2014). Perturbation of the circadian clock genes can potentially result in salivary gland disorders, such as chronic sialadenitis, Sjögren's syndrome or other complex immunological conditions (Papagerakis et al., 2014). It is recognized that circadian clock genes control cellular proliferation (Huang et al., 2011); hence, it is plausible that frequent perturbation of these genes may play a role in the cancer development. There are evidences suggesting that circadian clock genes are involved in regulation of expression of critical cancer-related genes, such as p53 and C-Myc (Fu et al., 2002; Hunt and Sassone-Corsi, 2007). Bmal1 and Per2 have been demonstrated to have tumor suppressor activities and alteration of their promoter methylation has been linked with poor clinical outcome and tumor progression in hematological malignancies and gliomas (Taniguchi et al., 2009; Xia et al., 2010). Preliminary evidence has been provided for alteration of the circadian clock genes in oral squamous cell carcinomas and salivary gland cancer (Papagerakis et al., 2014). Tang et al. demonstrated that Bmal1 as a tumor suppressor, increases the sensitivity of tongue squamous cell carcinoma (TSCC) to paclitaxel treatment, using TSCC cell lines and xenograft mouse models (Tang et al., 2017). In humans, oncogenes, such as p53 and Cyclin B1 have been reported to be direct targets of the circadian clock genes and to follow a circadian rhythm *in vivo* (Fu et al., 2002; Hunt and Sassone-Corsi, 2007). Furthermore, early studies in regard with the implications of clock genes in oral epithelium homeostasis, provided evidence of a rhythmic circadian expression profile of Per1, Cryl, and Bmal1; interestingly the major peak in Per1 expression in oral mucosa in healthy diurnal active volunteers coincided with p53 (a G1-phase marker) while the peak for Bmal1 coincided with cyclin B1 (a M-phase marker), in support of a circadian coordination of cell-cycle events in oral mucosa (Bjarnason et al., 2001; Gaucher et al., 2018), similar to other mucosa throughout the human gastro-intestinal tract (Buchi et al., 1991; Hoogerwerf, 2010). A recent review discusses the association of the circadian clock gene PER1 with oral cancer pathogenesis and suggested that changes in its expression may play an important role in the initiation and progression of oral squamous cell carcinoma (Nirvani et al., 2018).

Downregulation of hCRY2, hPER3 and hBMAL1 in tumoral tissues vs. their non-cancerous adjacent counterparts has been previously correlated with severity of tumor progression in a retrospective cohort of 40 head and neck squamous cell carcinoma patients (Hsu et al., 2012). A recent review article has further detailed the importance of circadian clock biology and its potential in various fields of dentistry (Janjic and Agis, 2019).

**Figure 1:**
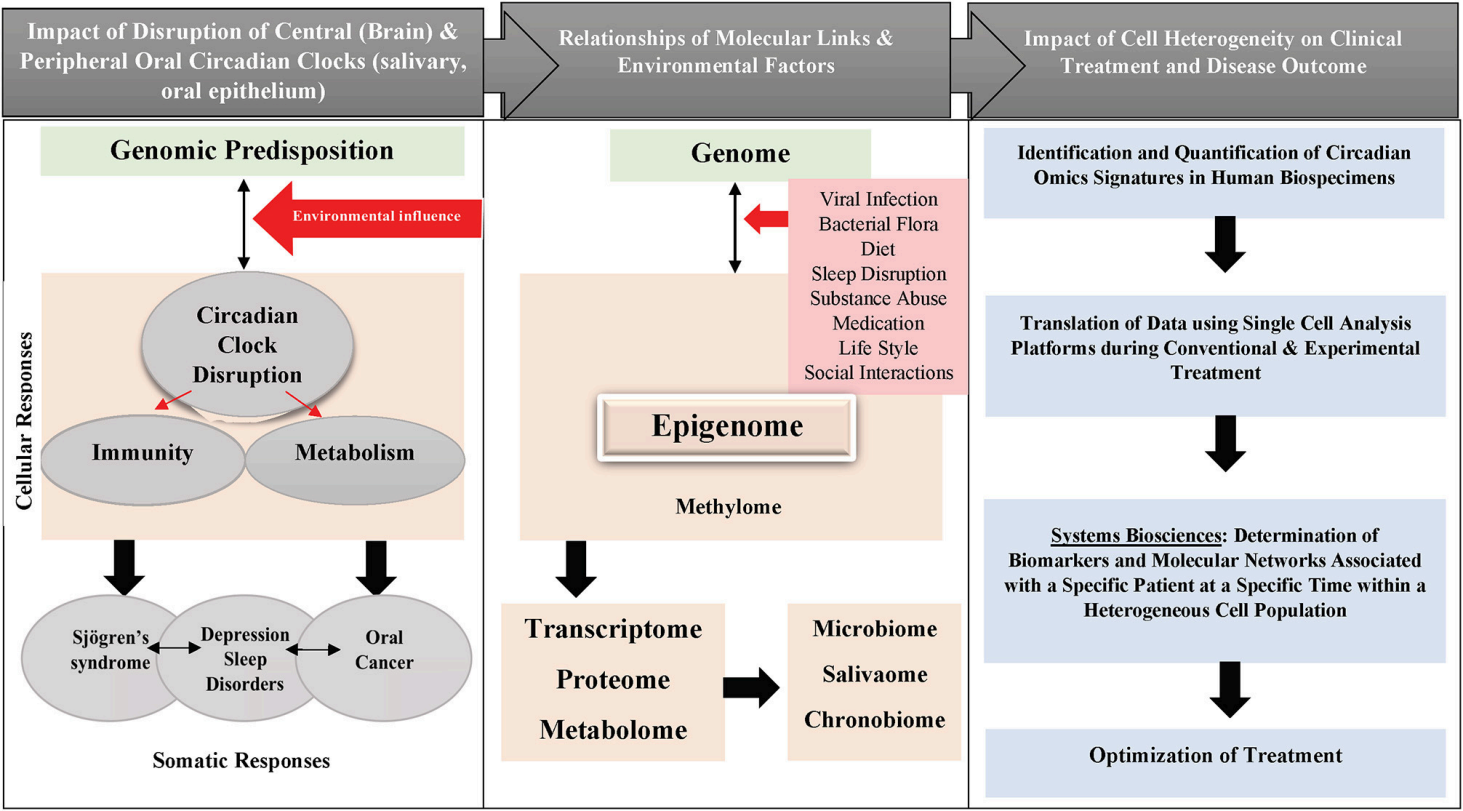
Schematic diagram showing the impact of circadian clock disruption on oral diseases pathogenesis and treatment.

Clock gene expression dysregulation in head and neck pathologies reported above is also summarized in Table 1. Taken all together, it's becoming evident that circadian clock disruption has major effects on oral diseases pathogenesis and treatment (Figure 1).

**Table 1:** Circadian gene expression profile and circadian gene defects in different head and neck pathologies.

**Circadian gene changes**	**Changes**	**Head and neck pathologies**	**Effect**	**Reference**
Clock genes and AQP5	Abnormal expression of clock genes	Sjögren's syndrome (SS) and chronic sialoadenitis	Xerostomia and salivary gland disorder	Papagerakis et al., 2014
Clock genes and SLC4A2/AE2, AQP5	Co-expression	Salivary gland tumors	Initiation and progression	Papagerakis et al., 2014
BMAL1	mTOR-driven upregulation	Oral cancer	Possible poor clinical outcome and tumor progression	Matsumoto et al., 2016
BMAL1	Alteration of tumor suppressor activity	Tongue squamous cell carcinoma	Increased sensitivity to paclitaxel treatment	Tang et al., 2017
PER1	Downregulation	Oral squamous cell carcinoma	initiation and progression	Nirvani et al., 2018
hCRY2, hPER3 and hBMAL1	Downregulation	Head and neck squamous cell carcinoma	Increases severity of tumor progression	Hsu et al., 2012
PER1 and CLOCK	Downregulation	Head and neck squamous cell carcinoma	Poor postoperative prognosis	Hsu et al., 2014
HS3ST2	Hypermethylation	Oral Cancer	Initiation and progression	Castilho et al., 2017

## Precision Oral Health, Multi-omics and System Biology Approaches

### Precision Health and Circadian Clock

The dominant paradigm of “one gene at a time” has historically been the core of biochemistry and molecular biology (Baggs and Hogenesch, 2010). However, the completion of the Human Genome Project (HGP) heralded a new era in the field of systems biology through its integrated big science approach (Shapiro, 1993; Hood and Rowen, 2013). This has resulted in an overarching need to integrate the data explosion emanating from digitalization of information, novel technologies with advanced bioinformatics tools, for meaningful applications in clinical medicine (Lopes et al., 2013; Boja et al., 2014). The full molecular cross-talk and pathway networks can now be captured/analyzed/deciphered using high throughput instrumentation in tandem with “state-of-the-art” computational tools in a systems biology manner. Traditional biochemical and molecular biology approaches have been useful in identifying basic underlying genetic components of the circadian clock, but progress toward a more robust understanding of the circadian clock global landscape has been impeded by the limited use of comprehensive/multi-omics approaches for clock gene/chronobiology studies (Chow and Kay, 2013). Using the Arabidopsis circadian clock model, Chow and Kay (2013), were able to demonstrate that the circadian clock is intricately regulated at multiple (from transcriptional to post-translational) levels. Significant enrichment of feedback loop motif in the predicted network was observed in a study focused on time-course gene expression datasets in rat suprachiasmatic nucleus, where circadian mechanisms were studied using a “reverse-engineering” approach to probe functional interactions among circadian genes by integrating various “omics” data (Wang et al., 2009). The wide spread use of omics tools will significantly improve our in-depth understanding of the circadian clock architecture in the systems biology pipeline, as well as provide mechanistic insight into the regulation of the circadian system within a given clinical context (Baggs and Hogenesch, 2010). Omics approaches (such as epigenomics, transcriptomics, and metabolomics) have also been used to study the regulation of the sleep-wake cycle, which is one of the vital daily physiologic rhythms (Goel, 2015).

### Oral Precision Health: Advances and Limitations in Omics Approaches and Circadian Clock Signaling Involvement

There is a complex, intricate and robust network of relationships between physiological output and the circadian clock, which requires novel technical and bioinformatics tools to unravel this complexity in a seamless manner (Doherty and Kay, 2010; Pruneda-Paz and Kay, 2010). In this complex network, cellular responses are coordinated by multiple pathways and as such the effect of a particular mutation can be compensated for by other pathways (Doherty and Kay, 2010). Molecular biology and genetics have advanced our knowledge of circadian control, but the mechanistic coordination of circadian rhythms by the circadian players still remain unclear. Deciphering the full complement of molecules (“omics” approach) and the crosstalk between multiple omics field (multi-omics approach) has the potential to provide a comprehensive insight into the complex circadian mechanism.

**Figure 2:**
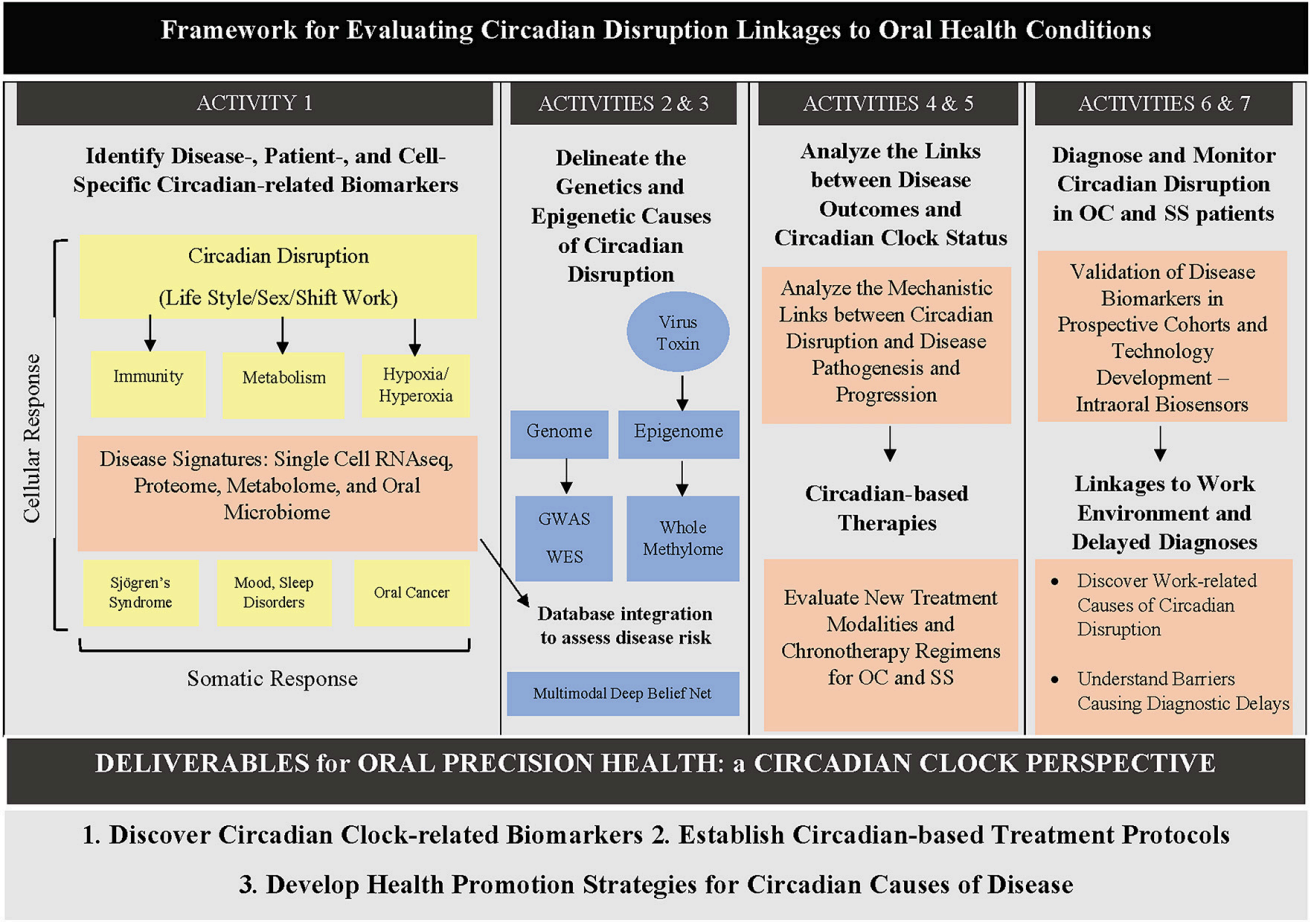
Diagram showing a proposed framework for evaluating circadian disruption links to oral health diseases. Different activities can be undertaken by oral health researchers to elucidate different parts of the circadian clock–oral health puzzle. GWAS: genome-wide association study; WES: whole exome sequencing.

Currently, there is a dearth of literature on the various high throughput circadian omics applied to oral health and head and neck pathologies. This gap is critical to address, given the evidence highlighting the major role circadian disruption plays in the development of oral, head and neck tissues as well as in their related diseases' pathogenesis. We discuss hereafter a few applications of omics-based approaches to studying circadian disruptions in head and neck pathologies, with an emphasis on oral, head and neck cancer (OC) and Sjögren's syndrome (SS). We also discuss known links of circadian clock signaling to HNSCC and SS. A potential framework for evaluating circadian disruption links to oral health diseases is provided for OC and SS (Figure 2).

#### Genomics

Genomics is a discipline of systems biology that addresses the full complement of genes in a genome (Shimeld et al., 2010). It also involves global studies of gene expression patterns under various conditions and stages of development (Barrett and Edgar, 2006). Using high density DNA microarrays, rhythmically expressed genes in mouse SCN and other tissues have been identified (Akhtar et al., 2002; Storch et al., 2002; Ueda et al., 2002; Sato et al., 2004). Out of hundreds of identified cycling genes, only about 50 cross-tissue cycling genes were found which, interestingly, included *Per2, Cry1, Bmal1*, and *Rev-erb*α (Sato et al., 2004). Sato et al. (2004), further carried out functional cell-based genomic analysis, to verify if a circadian transcription factor that activates Bmal1 expression could also be one of the candidate genes on this list of 50 cross-tissue cycling genes (Sato et al., 2004). They identified an orphan nuclear receptor, *Rora*, as a gene involved in activation of *Bmal1* transcription in the SCN, suggesting that antagonizing the activity of *Rora* and *Rev-erb*α (which also represses Bmal1) are crucial for maintenance of circadian clock function (Sato et al., 2004). In order to overcome the single platforms limitation of the genome-wide profiling to date published studies of head and neck squamous cell carcinomas (HNSCC), a recent multi-institution initiative coordinated by the US National Cancer Institute and National Human Genome Research Institute called “The Cancer Genome Atlas Network (TCGA)” has generated the today's most comprehensive integrative multi-platform genomic HNSCC profiling based of the analysis of 279 case cohort with well-annotated clinical information stratified by age, sex, race, anatomic site, stage, tumor histological differentiation, smoking and drinking status, human papillomavirus (HPV) infection, cancer treatment, clinical outcome and survival (Cancer Genome Atlas Network., 2015). By providing the HNSCC landscape of somatic mutations, DNA and RNA structural alterations, genome alterations and pathways through integrative bioinformatics analysis, the TCGA database proves an excellent starting point toward deciphering the HNSCC molecular and genomic heterogeneity, although the individual circadian profile, circadian disruption extent and comorbidity burden were not taken into account. A more careful analysis of the existing data, characterizing possible mutation or polymorphisms of clock genes and their targets in HNSCC, will further enhance our understanding of these pathologies.

#### Transcriptomics

Transcriptomics permits the large-scale examination of the full complement of RNA transcripts in a single cell or a population of cells (Manzoni et al., 2016). Mammalian transcriptome analysis using DNA microarrays has resulted in the identification of hundreds of tissue-specific circadian clock-controlled genes, resulting in the coordinated regulation of a vast array of biological processes (Delaunay and Laudet, 2002; Hsu and Harmer, 2014). Systems-level analysis of circadian mechanism permits differential comparisons of circadian transcript regulation between tissues, thus providing much needed insights into the roles, functions and regulation of the peripheral tissue-specific circadian clocks (Doherty and Kay, 2010). While measuring individual circadian rhythm disruption using cortisol, melatonin and body temperature can prove difficult in humans, transcriptomics has been successfully used in animal studies, which have identified novel biomarkers of circadian disruption in murine serum (Van Dycke et al., 2015). A more robust understanding of the circadian rhythm has been made possible throughout transcriptomics analysis of the suprachiasmatic nuclei, which has provided supporting evidence that the SCN transcription timing is controlled by mechanisms that reset the gating clocks (Pembroke et al., 2015). Diurnal variation of genome-wide transcriptional responses to 90 kilobecquerel (kBq) of Iodine-131 has been demonstrated in female BALB/c nude mice thyroid, suggesting that circadian rhythm may plays a role in response to irradiation of head and neck tumors (Langen et al., 2015, 2016). Although there is paucity of circadian genomics study of head and neck pathology, nine circadian clock genes have been investigated in patients with oral squamous cell carcinoma using quantitative real-time polymerase chain reaction and immunohistochemistry, of which PER1 and CLOCK were found differentially expressed in the peripheral blood of those patients and thus could serve as potentially reliable prognostic markers (Hsu et al., 2014). The same group reported that downregulation of BMAL1, CRY2, and PER3 expression was correlated with more advanced oral cancer stages (Hsu et al., 2012). PER1 and PER2 genes are considered to be tumor suppressor genes and their decreased expression has been reported in head and neck cancer (Uth and Sleigh, 2014). Using the golden hamster buccal mucosa, Tan et al. provided *in vivo* evidence for the differential expression of various circadian genes at different stages of cancer; demonstrating that prolonged circadian disruption leads to changes progressing through the stages of carcinogenesis (normal mucosa, precancerous lesion, and cancer) (Tan et al., 2015). New studies taking into account circadian transcripts expression differential disruption in HNSCC are needed to further understand the role of circadian clock transcription dysregulation in HNSCC.

#### Sequencing

Sequencing refers to various methods for the determination of the order of the nucleotides in nucleic acids (RNA or DNA) (Heather and Chain, 2016). This process, which was previously laborious and cumbersome, has now been up-scaled and automated such that identification of the nucleotide sequences at a genome-wide level has now become feasible at faster and affordable rates (Moorthie et al., 2011). This technology was very instrumental in the establishment of the Human Genome Project (HGP) (Shapiro, 1993), which has significantly impacted the precision medicine concept (Hood and Rowen, 2013). Also, the development of The Cancer Genome Atlas (TCGA) project has created a robust database that archives genomic profiles of over 500 cases of 20 different human cancer types (Chandran et al., 2016). Accuracy, speed and throughput depends on the generation of the sequencer (Heather and Chain, 2016). RNA sequencing (RNA-Seq) provides in-depth insight, global coverage and better resolution into the cell transcriptome, than traditional Sanger sequencing or microarray-based techniques (Kukurba and Montgomery, 2015). Various sequencing approaches have been extensively used to elucidate the pathophysiological roles of the circadian clock genes in Aedes albopictus, Drosophila Spp. and rats (Woon et al., 2006; Hughes et al., 2012; Summa et al., 2012; Kuintzle et al., 2017; Matsumura and Akashi, 2017), but sparsely used in humans due to disease heterogeneity, patient uniqueness, health care affordability, overwhelm of data which is left unanalyzed in the absence of adequate expertise, etc. Genomic sequencing has been used to identify the circadian gene CLOCK as mutated in other epithelial gastro-intestinal (GI)-derived cancers, such as colorectal cancer (Alhopuro et al., 2010). This demonstrates the potential of using sequencing approaches to investigate the role of circadian gene disruption in head and neck pathologies, particularly for oral cancer, salivary gland tumors, odontogenic tumors, etc.

#### Epigenomics

Epigenomics is the omics field that investigates the full complement of epigenetic changes, such as DNA methylation and histone modification in a cell or a population of cells (Sharma et al., 2010). Considering that the circadian clock system responds to environmental cues, there is burgeoning evidence that there is a plausible link between epigenomic regulators and the circadian clock (Pembroke et al., 2015). Epigenomics has been demonstrated to have predictive implications for resistance to therapy, risk stratification, tumor progression and resistance to therapy in head and neck cancer (Hsu et al., 2014; Langen et al., 2015, 2016). Dysregulation of DNA methylation of clock genes has previously been reported in diseased oral mucosa [reviewed in Papagerakis et al. (2014)]. The circadian gene CLOCK and histone-related genes, such as histone deacetylase HDAC3 and histone methyltransferase MLL3 play a role in regulating metabolism using histone modifications (Papagerakis et al., 2014). Castilho et al. reported that the gene heparan sulfate-glucosamine 3-sulfotransferase 2 (HS3ST2), which performs circadian functions, is frequently hypermethylated in oral cancer (Castilho et al., 2017). Heparan sulfate biosynthetic enzymes, such as HS3ST2, generate various biologically active heparan sulfate molecules. HS3ST2 has also been reported to be hypermethylated in various other human cancers affecting the colon, breast, stomach, prostate gland, lung and pancreas (Hwang et al., 2013). Studies have shown that inactivation mutation of the tumor suppressor gene phosphatase and tensin homolog (PTEN), in association with other epigenetic changes, leads to increased activity of phosphoinositide 3-kinase (PI3K)/AKT/mammalian target of rapamycin (mTOR) pathway, which is found in most squamous cell carcinomas of the head and neck region (Squarize et al., 2013). Furthermore, Matsumoto et al. demonstrated that mTOR signaling pathway and core clock protein BMAL1 can be activated by oxidation-driven loss of PTEN function (Matsumoto et al., 2016). Evidence indicated that circadian regulatory pathways may give rise to rhythmic epigenetic modifications resulting in circadian epigenomes and may result in malignant transformation of normal lung samples to non-small cell lung cancer (Salavaty, 2015). Hence, epigenomics is a vital tool for studying the role of circadian disruption in oral, head and neck pathologies.

#### Proteomics

Proteomics deals with large-scale study of the full complements of proteins in a cell, organism, tissue or body fluid (Graves and Haystead, 2002). This “omics” approach has been extensively used to study various pathologies in the head and neck area (Haigh et al., 2010; Garcia-Munoz et al., 2014; Gupta et al., 2015; Harris et al., 2015; Gleber-Netto et al., 2016; Khan et al., 2016; Li et al., 2016; Camisasca et al., 2017). Circadian clock genes have been reported to demonstrate 24h rhythms in salivary glands, and disruption of these clock genes may lead to autoimmune and inflammatory diseases affecting saliva production, such as the Sjögren syndrome (Zheng et al., 2012). Liquid chromatography coupled electrospray ionization (ESI) tandem mass spectrometry has been used to identify twelve novel proteins in minor salivary glands, which may indicate that salivary proteomes differences may be vital for specific oral functions (Siqueira et al., 2008). However, literature is lacking with respect to the use of proteomics to identify circadian clock disruption in head and neck pathologies, such as Sjögren syndrome.

#### Metabolomics

Metabolomics involves the use of high throughput evaluation of small molecular weight metabolites (which are downstream product of various metabolic processes in cells, tissues or body) to understand fundamental pathway changes in systems biology. The integration of high throughput tools like nuclear magnetic resonance (NMR) spectroscopy, liquid chromatography/mass spectrometry (LC/MS) and gas chromatography/mass spectrometry (GC/MS) into the omics field, has increased the accuracy of metabolite quantification with metabolomics (Weckwerth, 2003). It has been suggested that the application of integrated, systems-level, multi-omics approach can improve our understanding on the link between circadian rhythms and lipid metabolic networks (Eckel-Mahan and Sassone-Corsi, 2013; Ray and Reddy, 2016). Using a computational modeling, an integrated transcriptome-metabolome study demonstrated that there is synergism between nodes in specific metabolome pathways and circadian transcriptome (Eckel-Mahan et al., 2012). That study demonstrated that the uracil salvage pathway (which is a nucleotide salvage pathway that synthesizes nucleotides from intermediates in the degradative pathway for nucleotides) and other metabolic pathways, oscillates in a CLOCK-dependent circadian fashion. Using a drosophila model, Xu et al. demonstrated that disruption of the circadian rhythm by restricting feeding times results in disturbances of reproductive fitness (Xu et al., 2011). Also, a study has shown that *ad libitum* food intake (even at normal calories levels) is linked to obesity in mouse (Hatori et al., 2012; Kuroda et al., 2012; Joo et al., 2016). The human circadian metabolome has been described (Dallmann et al., 2012), and the efficacy of the use of metabolomics to study circadian rhythms has been largely investigated (Haigh et al., 2010; Gupta et al., 2015). Recently few studies are emerging linking metabolite profiles with HNSSC and SS (Bengtsson et al., 2016; Ishikawa et al., 2016, 2017; Ji et al., 2017; Sridharan et al., 2017; Lohavanichbutr et al., 2018; Mikkonen et al., 2018; Sant'Anna-Silva et al., 2018; Hsu et al., 2019). Interestingly one of the studies highlighted the importance of timing when evaluating the HNSCC metabolome (Ishikawa et al., 2017). Still the role of circadian metabolome in head and neck pathologies remain unexplored.

#### Lipidomics

Lipidomics permits the large-scale study of the total lipid complements in cells using analytic chemistry tools in systems biology (Yang and Han, 2016). Changes in the metabolism of thousands of lipid species is related to changes in numerous pathways and networks (Han, 2016). Circadian lipidomics is essential to unravel the link between lipid metabolism and circadian clock (Gnocchi et al., 2015). Using lipidomics, Loizides-Mangold et al. provided *in vitro* evidence that membrane lipid metabolites demonstrated oscillatory patterns in myotube cells and muscle tissue, which might impact insulin signaling (Loizides-Mangold et al., 2017). Such a finding is potentially of far reaching significance for future therapy for insulin resistance. Without yet linking circadian disruption and lipidomics profiles to HNSCC and SS few studies are pioneering the field of lipid biology and head and neck pathologies (Wang et al., 2017; Bednarczyk et al., 2019). Even though lipidomics has not been used to study circadian disruption in head and neck pathologies, it has been extensively used to study circadian control (Gooley and Chua, 2014; Adamovich et al., 2015; Gnocchi et al., 2015; Aviram et al., 2016; Han, 2016; Loizides-Mangold et al., 2017; Wang et al., 2017; Bednarczyk et al., 2019), promising new discoveries are waiting in the head and neck pathologies field.

#### Microbiomics

Microbiomics is a high throughput omics tool that provides in-depth understanding of the role of the microbiota on human health and physiology (Rajendhran and Gunasekaran, 2010). Recently, gut microbiome research has demonstrated a vital role for trillions of microorganisms resident in the gut as critical determinants of disease and health in the host (Liang and FitzGerald, 2017). Diurnal variations exists in gut microflora synchrony with the host circadian clock (Liang and FitzGerald, 2017); and the circadian clock plays a key role in regulating the microbiome and host responses to pathogens (Rosselot et al., 2016). Microbiomics has been used to study the circadian mechanism (Rosselot et al., 2016; Voigt et al., 2016; Liang and FitzGerald, 2017), and is a potentially useful mechanism for investigating circadian disruptions in head and neck pathologies with an emphasis on oral flora.

In addition to microbiomics, several studies examining the role of a large variety of ingested micro-organisms (viruses, bacteria etc), antigens, toxins and carcinogens, in oral cavity have highlighted the importance of stem cells differentiation which comprises a particularly pronounced circadian rhythms and a strong clock gene involvement in their equilibrium (Kellett et al., 1989; Bjarnason et al., 1999; Thomson et al., 1999; Janich et al., 2011). Alterations of both stem cell markers and clock genes have been detected in oral squamous cell carcinomas (Grimm et al., 2015). Approximately two thirds of oral cancers occur in the oral cavity (lip, tongue, floor of mouth, palate, gingival, alveolar and buccal mucosa, with tongue cancers considered the most aggressive), while the remainder occurs in the oropharynx (Shah and Batsakis, 2003). Evidence indicates infection of oral epithelial stem cells by high-risk types of human papillomavirus (HPV); clinical observations shown early lymphatic metastasis in HPV-related HNSCC (Desai et al., 2009; Albers et al., 2012). Deregulation of pathways controlling stem cell self-renewal (e.g., Wnt, Notch, Hedgehog, EGF, etc) leads to tumorigenesis in rodent models but also play a critical role in human oral, head and neck carcinogenesis (Winning and Townsend, 2000; Du et al., 2003; Nickoloff et al., 2003; Al-Hajj and Clarke, 2004; Klaus and Birchmeier, 2008). Both cancerous and normal stem cells are long-lived and thus can accumulate consecutive genetic changes under the continuous challenges imposed on the oral mucosa, the evolution of DNA methylation has allowed them to respond to environment cues in a flexible, yet stable manner to ensure their genetic stability, however it has been shown that variability in DNA methylation exists within HNSCC subtypes as well as same subtype interpatient variability, influenced by environmental factors, such as diet (Carvalho et al., 2006; Tan et al., 2008; Agrawal et al., 2011; Stransky et al., 2011; Sun et al., 2012; Colacino et al., 2013). Given that over 700 bacterial species inhabit the oral cavity, a growing body of evidence implicates human oral bacteria in the etiology of oral cancer (through potentially activation of alcohol and smoking-related carcinogens, locally or systematically through chronic inflammation), as well as an increased risk of these cancers in patients with periodontal disease and tooth loss (Ahn et al., 2012). The vast majority (90%) of oropharynx cancers seems likely related to high risk HPV (especially type 16) in contrast to only 5% of the oral cavity cancers (Marani and Heida, 2018). The TCGA network have revealed novel and previously recognized gene and chromosomal region copy number alterations, mutations, and expression clusters and defined their frequency, co-occurrence, and relationship to common and rare subtypes of HPV-negative and HPV-positive tumors that vary in prognosis (Cheng et al., 2018). Furthermore, a recent study undertook a comprehensive genomic and transcriptomic characterization of long-established 15 HPV-negative and 11 HPV-positive head and neck squamous cell carcinoma (HNSCC) lines, proven valuable as *in vitro* HNSCC experimental models, comparatively with the genomic alterations of the 279 HNSCC tumors from the TCGA database; the findings highlighted the importance of a network of pathways and the combined contribution of genomic and transcriptomic alterations (shared or HNSCC subtype-specific and HPV status-related) in promotion of the malignant phenotype and therapeutic resistance, which was heretofore limited to selected gene candidates (Cheng et al., 2018). Other studies have undertaken a comprehensive molecular multi-layered omics-based approach applied to a single HNSCC-derived cell line and its single-cell derived subclones, to elucidate the tumor heterogeneity and treatment-induced clonal selection (Niehr et al., 2018) or a single signaling pathway (e.g., AKT/PKB) (Fruman et al., 2017; Manning and Toker, 2017). For instance, Niehr et al. (2018), analyzed the HNSCC-derived HPV-negative cell line FaDu, recognized as an *in vitro* HNSCC model of cisplatin resistance, by using targeted next generation sequencing, fluorescence *in situ* hybridization, microarray-based transcriptome and mass spectrometry-based phosphoproteome analysis. This was followed by siRNA-based gene silencing to determine the causal relationship between molecular features and resistant phenotypes, and the clinical relevance of molecular findings was validated throughout survival analysis of the TCGA dataset of HPV-negative HNSCC patients with recurrent disease after cisplatin-based chemo-radiation; their findings demonstrated a link between the intratumor heterogeneity and clonal evolution as mechanisms of drug resistance in HNSCC and established mutant gain-of-function *TP53* variants and the PI3K/mTOR pathway as molecular targets for treatment optimization in HNSCC (Niehr et al., 2018). Interestingly we have shown that mTOR pathway is directly linked to clock genes signaling (Matsumoto et al., 2016). Additional studies are needed to further explore the links between circadian clock signaling, HNSCC and microbiome of oral cavity.

## Future Perspectives in Precision Oral Health and Circadian Biology

### Saliva as a Source of Multi-Omics and Circadian Clock Disruption Profiles

More recently, an increasing interest has been noted in the incorporation of saliva analyses (metabolomics, biomarkers profiling, microbiome, etc.) in the oral cancer diagnostics and progression prediction, while the future of salivary diagnostics seems to rely heavily on the integration of all the omics (Mikkonen et al., 2016; Washio and Takahashi, 2016; Kaczor-Urbanowicz et al., 2017).

More recently we are assisting to the circadiOmics and chronobiome emergence which integrates high-throughput time series of tissue and condition-specific circadian genomics, transcriptomics, proteomics and metabolomics by taking into account the 24 h expression profiling variations, thus highlighting the critical importance of a systems approach to achieve a more accurate view of the underlying global network under circadian regulation as well as standardization of the time of specimen collection and of the experimental measurements; CircadiOmics uses data integration and artificial intelligence module to compile information from all the currently available databases, web services and tools which are regularly updated; users can search the high-throughput experimental data interactively plot time courses across different conditions and tissues (Eckel-Mahan et al., 2012; Patel et al., 2012). Saliva in fact may be the biological fluid of excellence to study the effects of circadian clock disruption in head and neck pathologies using a multi-omics approach combined with multiple saliva sampling.

### Single Cell Circadian Profile in Oral Tissues: Opportunities and Pitfalls

Single cell analysis of circulating tumor cells and of tumor biopsies for carcinomas has been applied both in cell lines and clinical samples leading to enhanced diagnostic and therapeutic tools. No studies that include single cell analysis have been published in the area of head and neck pathologies yet (Stucky et al., 2017). On the other hand, single cell analysis is just emerging in the field of circadian biology (Abraham et al., 2018). Analyzing the extend circadian rhythms disruption at the single cell level and correlating the level of disruption with clinical outcomes will offer us the highest possible level of knowledge on the role of circadian clock disruption in head and neck pathologies. However, the amount of data and the correct interpretation for use in clinical scenarios may be delayed due to complexity of both single cell analyses and the lack of precise knowledge of circadian clock system disruption links to oral diseases.

## Conclusion

**Figure 3:**
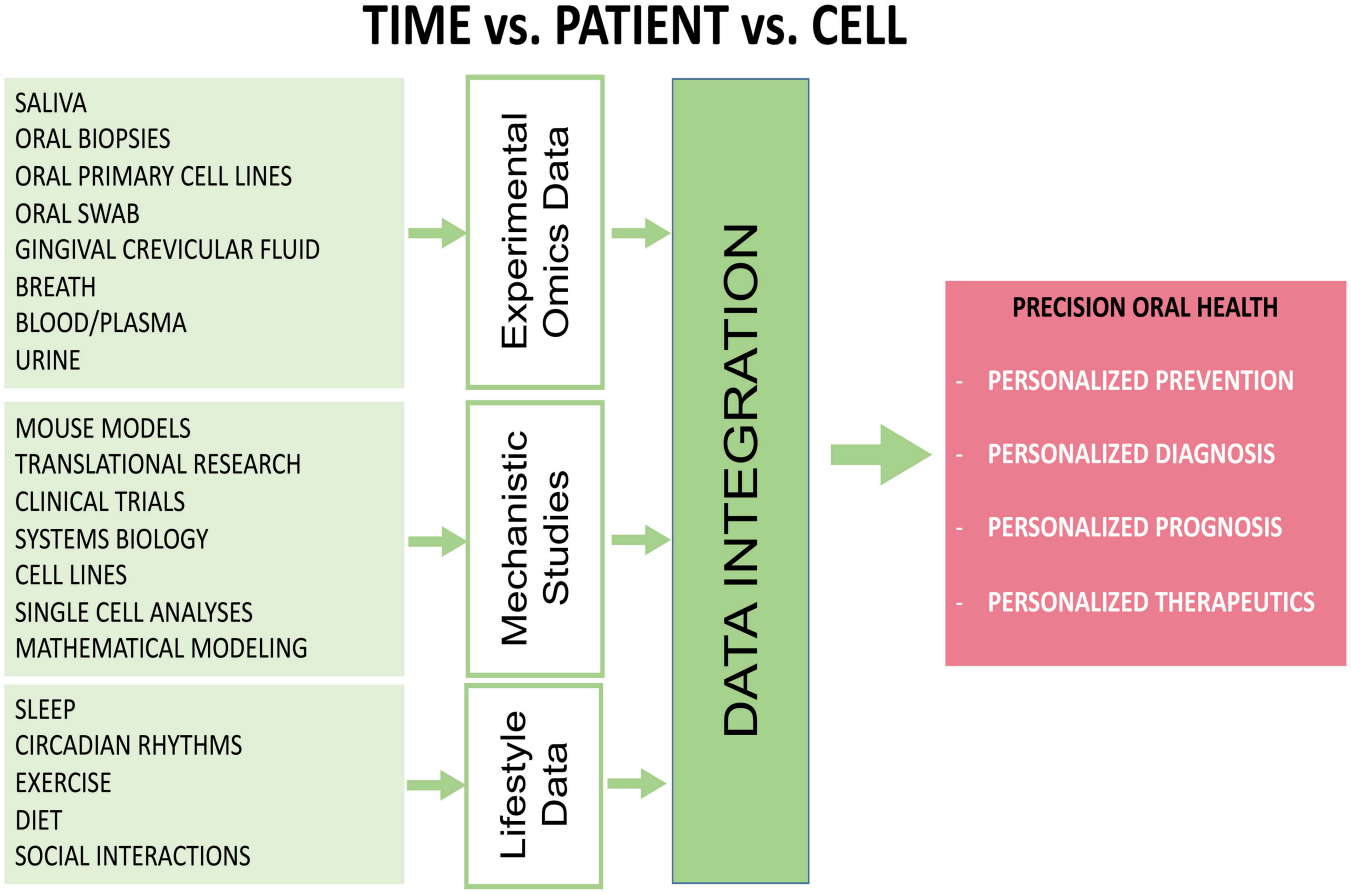
Diagram showing that applications of various high-throughput omics-based approaches and data integration are necessary to study the role of circadian clock disruption in head and neck pathologies. Data can be collected from biological fluids and from components of patient lifestyle and combined with mechanistic data can provide a holistic view of a patient's health. This approach would lead to better prevention, diagnosis, targeted therapies and hopefully policy changes that will result in better outcomes overall.

Most head and neck pathologies show a broad cellular heterogeneity making it difficult to achieve an accurate diagnosis and efficient treatment (Graf and Zavodszky, 2017; Lo Nigro et al., 2017). Single cell analysis of circadian omics (Lande-Diner et al., 2015; Abraham et al., 2018), may be a crucial tool needed in the future to fully understand the circadian control of head and neck diseases. It becomes more obvious that there is only a small genetic component but a largely unknown epigenetics and/or environmental component for most of the head and neck pathologies (Moosavi and Motevalizadeh Ardekani, 2016; Hema et al., 2017; Lindsay et al., 2017). Exposure to bacteria, virus, toxins, pesticides, stress, life styles changes, sleep disorders, etc, all (in combination or alone) affecting the circadian clock and increase vulnerability to head and neck pathologies (Hooven et al., 2009; LeGates et al., 2014; Cui et al., 2016; Potter et al., 2016; Koch et al., 2017; Kopp et al., 2017). Omics approaches need to be combined with tests and biosensor data for each patient to get most out of them. Recently, there was noted a growing interest on clinical testing of the ability of drugs/small molecules to reset the circadian clock and treat diseases, such as diabetes and cancer (Antoch and Chernov, 2009; He and Chen, 2016; Tamai et al., 2018). Combined omics will be needed to monitor the effects of drugs and individual health outcome. Not least, saliva may become a gold standard for circadian time points monitoring and continuous assessment, and may provide information on protein, bacteria, toxins, stress-induced cortisol, and other circadian biomarkers compared to saliva melatonin, an indicator of the circadian clock (Novakova et al., 2011; Shinohara and Kodama, 2011). Many omics-based techniques are yet to be widely utilized for studying the effect of circadian disruption in diseases emanating from the head and neck regions. Considering that the circadian rhythms controls a vast repertoire of metabolic and physiological activities in the body, it is vital to decipher circadian oscillations using novel, “state-of-the-art,” high throughput “omics-based tools.” It is also important to use these tools in an integrated multi-omics manner, rather than in isolation, and most importantly within a well-defined personalized clinical context which takes in account individual variability, disease predisposition, lifestyle, environmental exposure, the very core of the oral precision medicine etc. Equally important, a comprehensive multi-omics map of circadian network (Patel et al., 2012) which integrates circadian, transcriptomics, metabolomics, genomics and proteomics has been suggested as necessary step toward precision health. Another study which generated high-resolution multi-organ expression data, showed that almost 50% of the mouse genome oscillate with circadian rhythm (Zhang et al., 2014) further supporting the importance of circadian clock in precision health approaches. Cyclic ordering by periodic structure (CYCLOPS) machine-learning algorithm has also been used to identify rhythmic transcripts in human lung and liver, as well as hundreds of drug targets and diseases-related genes; this method has been validated using mouse and human data (Anafi et al., 2017). It is envisaged that such a map will provide circadian clock researchers with access to mining of high-resolution multi-omics data from the repository. With increasing use of “omics” techniques for circadian clock biology, multidimensional knowledge of the architecture and functioning of the circadian system would be generated. Application of novel technologies, such as single cell technology and metagenomics is poised to significantly improve our knowledge of circadian biology. Not least, greater understanding of many more physiological and pathological conditions in the head and neck region would be acquired thought data integration approaches (Figure 3).

## Author Contributions

HA and PP conceptualized, designed, prepared, and critically revised the manuscript and figures. SP was involved in the design, and critical intellectual revision of the paper. All authors were involved in preparing the manuscript and had final approval of the submitted version.

### Conflict of Interest Statement

The authors declare that the research was conducted in the absence of any commercial or financial relationships that could be construed as a potential conflict of interest.
